# PUBLIC SECTOR INVOLVEMENT IN AGE-FRIENDLY CITIES AND COMMUNITIES: A SCOPING REVIEW

**DOI:** 10.1093/geroni/igad104.1508

**Published:** 2023-12-21

**Authors:** Emily Greenfield, Natalie Pope, Laura Keyes, Elizabeth Russell

**Affiliations:** Rutgers, The State University of New Jersey, New Brunswick, New Jersey, United States; Rutgers Social Work, New Brunswick, New Jersey, United States; University of North Texas, Denton, Texas, United States; Trent University, Peterborough, Ontario, Canada

## Abstract

The global age-friendly cities and communities (AFCC) movement has long centered the involvement of the public sector, calling on high-ranking authorities to commit to improving the built, social, and service environments of their localities. However, there is little understanding of how the public sector is involved in actual practice. To address this gap, we conducted a scoping review of peer-reviewed articles published since 2010, with a focus on studies in Canada and the United States, to synthesize the evidence on the ways in which the public sector has been involved with AFCC work. Our review identified four primary themes. The first theme describes the variety across studies in descriptions of the overall positioning of the public sector in AFCC efforts, with public sector actors ranging from leaders to partners to targets for advocacy. The second theme addresses activities that are initiated by a public administration that targets age-friendly improvements for that administration. The third theme encompasses activities that help to connect a public administration with, as well as influence, outside actors, primarily the private sector and government administrations at other systems levels. The final theme elucidates the various ways in which the public sector is involved with AFCC assessment, evaluation, and research. We discuss the implications of our findings for practice, policy, and continued empirical research. We also discuss theoretical implications for AFCC efforts in terms of distinguishing “whole of government” from “whole of society,” grassroots, and planning-specific approaches.

